# Infant Exposure to Dolutegravir Through Placental and Breast Milk Transfer: A Population Pharmacokinetic Analysis of DolPHIN-1

**DOI:** 10.1093/cid/ciaa1861

**Published:** 2020-12-21

**Authors:** Laura Dickinson, Stephen Walimbwa, Yashna Singh, Julian Kaboggoza, Kenneth Kintu, Mary Sihlangu, Julie-Anne Coombs, Thokozile R Malaba, Josaphat Byamugisha, Henry Pertinez, Alieu Amara, Joshua Gini, Laura Else, Christie Heiberg, Eva Maria Hodel, Helen Reynolds, Landon Myer, Catriona Waitt, Saye Khoo, Mohammed Lamorde, Catherine Orrell, DolPHIN-1 Study Group, Ritah Nakijoba, Isabella Kyohairwe, Johnson Magoola, Emmanuel Ssempija

**Affiliations:** 1 Department of Molecular & Clinical Pharmacology, University of Liverpool, Liverpool, United Kingdom; 2 Infectious Diseases Institute, Makerere University College of Health Sciences, Kampala, Uganda; 3 Desmond Tutu HIV Foundation, University of Cape Town, Cape Town, South Africa; 4 Division of Epidemiology & Biostatistics, School of Public Health & Family Medicine, University of Cape Town, Cape Town, South Africa

**Keywords:** dolutegravir, population pharmacokinetics, pregnancy, breast milk, infant pharmacokinetics

## Abstract

**Background:**

Rapid reduction in human immunodeficiency virus (HIV) load is paramount to prevent peripartum transmission in women diagnosed late in pregnancy. We investigated dolutegravir population pharmacokinetics in maternal plasma, umbilical cord, breast milk, and infant plasma samples from DolPHIN-1 participants (NCT02245022) presenting with untreated HIV late in pregnancy (28–36 weeks gestation).

**Methods:**

Pregnant women from Uganda and South Africa were randomized (1:1) to daily dolutegravir (50 mg/d) or efavirenz-based therapy. Dolutegravir pharmacokinetic sampling (0–24 hours) was undertaken 14 days after treatment initiation and within 1–3 weeks after delivery, with matched maternal and cord samples at delivery. Mothers were switched to efavirenz, and maternal and infant plasma and breast milk samples were obtained 24, 48, or 72 hours after the switch. Nonlinear mixed-effects modeling was used to describe dolutegravir in all matrices and to evaluate covariates.

**Results:**

A total of 28 women and 22 infants were included. Maternal dolutegravir was described by a 2-compartment model linked to a fetal and breast milk compartment. Cord and breast milk to maternal plasma ratios were 1.279 (1.209–1.281) and 0.033 (0.021–0.050), respectively. Infant dolutegravir was described by breast milk–to–infant and infant elimination rate constants. No covariate effects were observed. The median predicted infant dolutegravir half-life and median time to protein-adjusted 90% inhibitory concentration (0.064 mg/L) for those above this threshold were 37.9 (range, 22.1–63.5) hours and 108.9 (18.6–129.6) hours (4.5 [0.8–5.4] days) (n = 13), respectively.

**Conclusions:**

Breastfeeding contributed relatively little to infant plasma exposure, but a median of 4.5 days of additional prophylaxis to some of the breastfed infants was observed after cessation of maternal dolutegravir (3–15 days postpartum), which waned with time postpartum as transplacental dolutegravir cleared.

## Teaser

The population pharmacokinetic model described dolutegravir in maternal and infant plasma and transplacental and breastmilk passage. Based on infant elimination profiles, dolutegravir provided a median of 4.5 days prophylactic coverage to breastfed infants following maternal drug cessation 3-15 days postpartum.

Annually, approximately 1.5 million women living with human immunodeficiency virus (HIV) worldwide become pregnant, mostly in low- or middle-income countries. Mother-to-child transmission and increased infant mortality rates are significantly associated with late initiation of antiretroviral therapy (ART; ≥28 weeks gestation) [1]. Safe and effective ART that quickly reduces viral load is paramount to prevent peripartum transmission.

The World Health Organization recently updated ART guidelines to recommend dolutegravir-based combination therapy as the preferred first-line regimen for adults living with HIV [2], including pregnant women and women of child-bearing potential. Although initial safety concerns regarding neural tube defects within the first trimester of pregnancy were reported [2], no safety signals have been observed when dolutegravir treatment is started later in pregnancy [3, 4]. Advantages of dolutegravir over efavirenz-based therapy include lower drug-drug interaction potential, higher genetic barrier to resistance, and more rapid onset of viral load reduction [5, 6].

DolPHIN-1 (Dolutegravir in pregnant HIV mothers and their neonates; NCT02245022), an open-label, randomized, clinical study in pregnant women presenting with undiagnosed HIV late in pregnancy (28–36 weeks gestation) demonstrated superior virological response of dolutegravir-based therapy compared with the efavirenz-based standard of care (SoC). There was a significant difference in time to HIV RNA load <50 copies/mL and the proportion of women with undetectable viral load at 2 weeks postpartum [7]. These findings were confirmed in the primary analysis of DolPHIN-2, a larger study [8]. Moreover, similar maternal dolutegravir plasma exposures during the third trimester and postpartum were observed in DolPHIN-1, with detectable infant dolutegravir concentrations 1–3 days after maternal dolutegravir was stopped [7].

The current analysis aimed to develop a population pharmacokinetic model to describe dolutegravir disposition in maternal plasma (ante and postpartum), umbilical cord, breast milk, and plasma of breastfed infants 1–3 days after cessation of maternal dolutegravir treatment and to evaluate potential covariate effects in mothers and their infants enrolled in DolPHIN-1.

## METHODS

### Study Design and Pharmacokinetic Sampling

The DolPHIN-1 (NCT02245022) study design has been described elsewhere [7]. Briefly, HIV-1–infected pregnant women with HIV diagnosed in late pregnancy (≥28–36 weeks gestation), who had not taken ART within the last 6 months and had never received integrase inhibitors, were eligible for the study. Individuals were screened at antenatal clinics associated with study sites in Uganda (Infectious Disease Institute, Kampala) and South Africa (Desmond Tutu Health Foundation Clinical Trials Unit, Cape Town). Excluded were patients with active hepatitis B, history of unstable liver conditions, abnormal laboratory parameters, pregnancy-associated abnormalities (eg, severe preeclampsia), or psychiatric illnesses [7].

The Joint Clinical Research Centre Research Ethics Committee (Uganda), the University of Cape Town Human Research Ethics Committee (South Africa), and the University of Liverpool Ethics Committee (United Kingdom) provided ethical approval. Women gave written informed consent [7].

To comply with national guidelines [9, 10] women commenced SoC ART on the day of HIV diagnosis, while screening was underway. Eligible patients were randomized (1:1) to dolutegravir-based (50 mg/d) or efavirenz-based ART with a nucleoside reverse-transcriptase inhibitor backbone of tenofovir disoproxil fumarate plus emtricitabine or lamivudine. Intensive dolutegravir pharmacokinetic sampling over 24 hours was performed 14 days after therapy initiation (third trimester) and within 2 weeks after delivery (postpartum). Where possible, paired maternal plasma and cord samples were taken at delivery. Breast milk and infant plasma were obtained postpartum, 2–6 and 24 hours after the final dolutegravir dose in all mother-infant pairs. Women switched to SoC and randomized to paired maternal and infant plasma and breast milk sampling at 48, 72, or 96 hours after the switch (Supplementary Figure 1). Dolutegravir concentrations were quantified using validated liquid chromatography with tandem mass spectrometry methods [11, 12], with a lower limit of quantification (LLQ) of 0.01 mg/L for all matrices.

### Population Pharmacokinetic Modeling

Nonlinear mixed-effects modeling (NONMEM, version 7.4, ICON Development Solutions) [13] was used to simultaneously describe dolutegravir in maternal plasma, cord, and breast milk (maternal model) and to describe dolutegravir in infants using a sequential approach. Covariates were investigated to describe variability in maternal and infant pharmacokinetics. Modeling methods are outlined in the Supplementary Material.

### Dolutegravir Intercompartmental Exposure Ratios, Time to In Vitro Protein-Adjusted 90% Inhibitory Concentration, and Relative Infant Dose

Dolutegravir pharmacokinetic profiles were simulated for maternal plasma, cord, and breast milk and for infants using individual predicted parameters to calculate the area under the curve over 24, 48, 72, and 96 hours (AUC_0–24,_ AUC_0–48_, AUC_0–72_, AUC_0–96_) after the final postpartum dose. Dolutegravir AUC ratios of cord–maternal plasma at delivery (at 0–24 hours) and breast milk–maternal plasma and infant–maternal plasma (at 0–24, 48, 72, and 96 hours) were determined. The infant half-life was calculated as ln(2)/*k*_INF_, where *k*_INF_ is the infant elimination rate constant. The model was also used to predict time to the in vitro protein-adjusted (PA) 90% inhibitory concentration (IC_90_) of 0.064 mg/L [14] for infants.

The dolutegravir infant dose was estimated as follows: Infant dose (mg/kg/d) = breast milk concentration (mg/L) × volume ingested (L/kg/d). The relative infant dose (RID) from breast milk was estimated as follows: RID (%) = [(infant dose/kg)/(maternal dose/kg)] × 100. For the volume of breast milk ingested, a value of 0.15 L/kg/d is widely assumed for pharmacokinetic analyses [15–17].

## RESULTS

### Patients and Pharmacokinetic Sampling

Of 29 women randomized to receive dolutegravir, 28 were included in the maternal model (1 was withdrawn owing to nonadherence). Twenty-seven women had paired third trimester and postpartum visits; another participant withdrew before the postpartum visit owing to multiclass drug resistance, which was not integrase inhibitor related and achieved virological suppression once switched to a dolutegravir plus darunavir/ritonavir-containing regimen. Nineteen women had plasma samples obtained at delivery, and 17 cord samples were available, with 1 woman excluded owing to suspected nonadherence (values below the LLQ in plasma and cord samples) [7]. A total of 533 maternal plasma (250 antepartum, 18 delivery, and 265 postpartum samples), 16 cord, and 80 breast milk samples were used to develop the maternal model.

Of 27 infants, 22 had a recorded delivery time and could be included in the model (65 samples). The initial dolutegravir dose received by infants through transplacental passage was 39.9 mg (range, 15.5–59.0 mg), determined by multiplication of cord concentration and maternal central volume of distribution, corresponding to 12.5 mg/kg birth weight (5.0–19.6 mg/kg). Maternal and infant demographics are summarized (Table 1). Three of 533 maternal plasma (0.6%; 1 delivery and 2 postpartum samples) and 1 of 65 infant plasma samples (1.5%) had concentrations below the LLQ and were included as LLQ/2. The 39% of breast milk samples below the LLQ (31 of 80 samples) were included using the M3 method in NONMEM software.

**Table 1.** Demographic and Other Characteristics of Mothers and Infants Included in the Maternal and Infant Dolutegravir Population Pharmacokinetic Models

Parameter	Value
Mothers (n = 28)	
Study site, no. (%)	
Uganda	14 (50)
South Africa	14 (50)
Baseline age, median (range), y	27 (19–42)
Baseline weight, median (range), kg	67 (44–160)
Mode of delivery, no. (%)	
Known vaginal delivery	23 (82)
Cesarean delivery	3 (11)
Data missing	2 (7)
Postpartum sampling interval, median (range), d	7 (2–18)
Postpartum sampling interval, no. (%)	
Within 1 wk	15 (56)
Within 2 wk	9 (33)
Within 3 wk	3 (11)
Infants (n = 22)	22
Sex, no. (%)	
Male	17 (77)
Female	5 (23)
Study site, no. (%)	
Uganda	10 (45)
South Africa	12 (55)
Median value (range)	
Birth weight, kg	3.3 (2.5–4.3)
Body surface area, m^2^	0.22 (0.18–0.25)
Postmenstrual age, wk	40 (36–44)
Gestational age, wk	39 (35–43)
Postnatal age, d	7 (3–18)

### Population Pharmacokinetic Modeling

**Figure 1.**
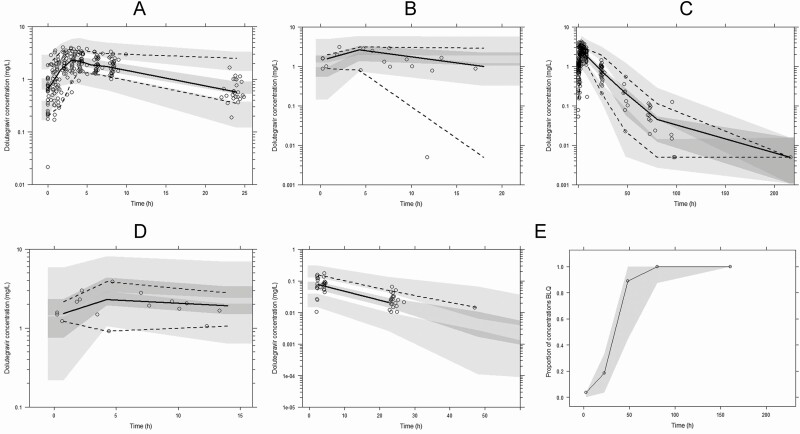
Dolutegravir visual predictive check for maternal plasma at third trimester (n = 28) (*A*), maternal plasma at delivery (n = 18) (*B*), maternal plasma postpartum (n = 27) (*C*), umbilical cord (n = 16) (*D*), and breast milk (n = 27) (*E*), along with the proportion below the lower limit of quantification (LLQ). Maternal plasma postpartum and breast milk samples were obtained after the final maternal dolutegravir dose. Lines represent percentiles of the observed data (fifth, 50th, and 95th percentiles), and shaded areas, 95% confidence intervals of the simulated data. Observed concentration-time data for maternal plasma (250 concentrations for the third trimester, 18 at delivery, and 265 postpartum), umbilical cord (16 concentrations), and breast milk (80 concentrations) are superimposed (*open circles*).

**Figure 2.**
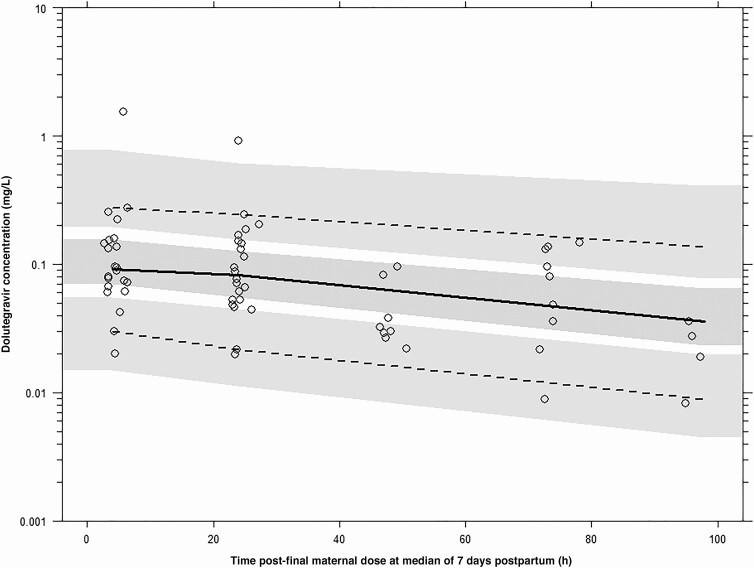
Dolutegravir prediction-corrected visual predictive check for infant plasma (n = 22) after the final maternal dolutegravir dose, given a median of 7 days postpartum. Lines represent percentiles of the observed data (fifth, 50th, and 95th percentiles), and shaded areas, 95% confidence intervals of the simulated data. Observed concentration time data are superimposed (65 concentrations [*open circles*]).

**Figure 3.**
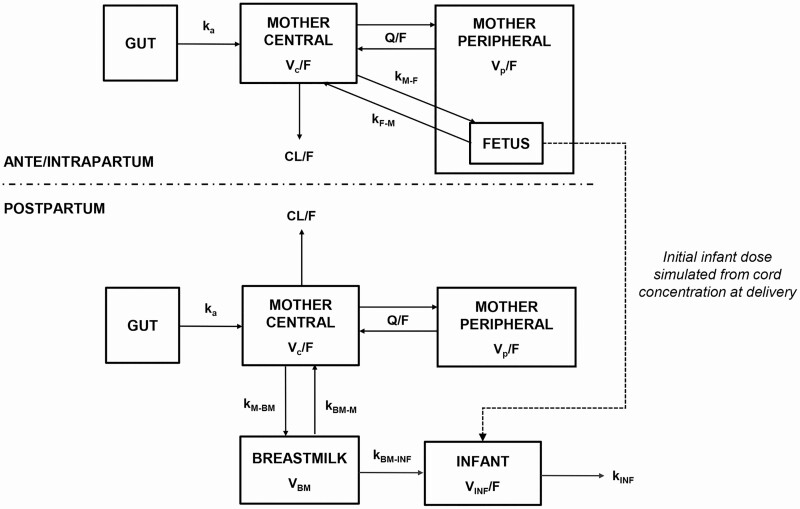
Schematic of the population pharmacokinetic model to simultaneously describe dolutegravir in maternal plasma, umbilical cord, and breast milk. Maternal plasma, cord and breast milk individual model estimates were then fixed to describe dolutegravir pharmacokinetics in infant plasma using a sequential approach. Abbreviations: CL/F, apparent oral clearance; *k*_a_, absorption rate constant; *k*_BM-INF_, breast milk–to–infant transfer rate constant; *k*_BM-M_, breast milk–to–mother transfer rate constant; *k*_F-M_, fetus-to-mother transfer rate constant; *k*_INF_, infant elimination rate constant; *k*_M-BM_, mother–to*–*breast milk transfer rate constant; *k*_M-F_, mother-to-fetus transfer rate constant; Q/F, intercompartmental clearance; RSE, relative standard error; V_BM_, volume of the breast milk compartment; V_c_/F, apparent volume of distribution of the central compartment; V_INF_/F, infant apparent volume of distribution; V_p_/F, volume of the peripheral compartment.

Dolutegravir concentrations for the maternal and infant models are presented in Figures 1 and 2, respectively, and schematics of the maternal and infant model structures are shown in Figure 3. Dolutegravir pharmacokinetics in maternal plasma were described by a 2-compartment model parameterized by apparent oral clearance (CL/F), apparent volume of distribution of the central compartment (V_c_/F) and peripheral compartment, intercompartmental clearance, and the absorption rate constant (*k*_a_). The central compartment was linked to a fetal compartment of negligible volume (which did not alter the maternal compartment) and a breast milk compartment of fixed volume (0.125 L [16, 18]) by first-order processes (mother-to-fetus, fetus-to-mother, mother–to–breast milk, and breast milk–to–mother transfer rate constants)

Infant dolutegravir was described by a 1-compartment model in which the initial dose was through transplacental transfer, followed by input over time from breast milk. Infant dolutegravir disposition was parameterized by an input transfer rate constant from the breast milk, an infant elimination rate constant (*k*_INF_), and the infant apparent volume of distribution. Interindividual variability was included for CL/F, V_c_/F, and *k*_INF_, and interoccasion variability for CL/F. Proportional error models were used throughout.

After univariable analysis, only days postpartum on CL/F generated a significant decrease in objective function value (−7.34); no covariates had a significant association with V_c_/F. Days postpartum on CL/F did not remain in the model after backward elimination. Covariates were not evaluated for the fetal or breast milk compartments as interindividual variability could not be estimated for the transfer rate constants. No infant covariates were associated with *k*_INF_. Model parameters, visual predictive check, and prediction-corrected visual predictive check for the maternal and infant model, respectively are presented in Table 2 and Figures 1 and 2. Goodness-of-fit plots are also shown (Supplementary Figure 2).

**Table 2.** Population Pharmacokinetic Parameter Estimates and Relative Standard Errors Determined From the Final Simultaneous Model for Mothers (Maternal Plasma, Umbilical Cord, and Breast Milk) and the Sequential Model for Infants Included in DolPHIN-1

Parameter	Estimate (RSE%)a
Maternal plasma (n = 28)	
CL/F,^b^ L/h	1.50 (1.9)
V_c_/F,^b^ L	24.6 (4.7)
Q/F, L/h	0.0138 (3.7)
V_p_/F, L	2.01 (16.7)
*k*_a_, h^−1^	0.75 (4.9)
Correlation between CL/F and V_c_/F	0.88 (23.4)
Umbilical cord (n = 16)	
*k*_M-F_, h^−1^	2.81 (3.6)
*k*_F-M_, h^−1^	2.20 (40.9)
Breast milk (n = 27)	
*k*_*M*-BM_, h^−1^	0.0027 (32.3)
*k*_BM-M_, h^−1^	16.3 (4.6)
V_BM_, L	0.125 (Fixed)
Infant (n = 22)	
*k*_BM-INF_, h^−1^	3.22 (16.2)
*k*_INF_,^b^ h^−1^	0.0162 (6.0)
V_INF_/F, L	30.1 (21.3)
Residual error, %	
Maternal plasma	36.5 (0.3)
Umbilical cord	33.0 (0.4)
Breast milk	58.4 (0.5)
Infant	34.4 (22.1)

Abbreviations: CL/F, apparent oral clearance; *k*_a_, absorption rate constant; *k*_BM-INF_, breast milk–to–infant transfer rate constant; *k*_BM-M_, breast milk–to–mother transfer rate constant; *k*_F-M_, fetus-to-mother transfer rate constant; *k*_INF_, infant elimination rate constant; *k*_M-BM_, mother–to*–*breast milk transfer rate constant; *k*_M-F_, mother-to-fetus transfer rate constant; Q/F, intercompartmental clearance; RSE, relative standard error; V_BM_, volume of the breast milk compartment; V_c_/F, apparent volume of distribution of the central compartment; V_INF_/F, infant apparent volume of distribution; V_p_/F, apparent volume of the peripheral compartment.

^a^RSE is calculated as (standard error of estimate/estimate) × 100.

^b^The interindividual variability was 14.3% (RSE, 6.0%) for CL/F, 20.7% (23.7%) for V_c_/F, and 43.6% (61.6%) for *k*_INF_ , and the interoccasion variability was 21.0% (RSE, 0.6%) for CL/F.

### Dolutegravir Intercompartmental Exposure Ratios, Time to PA-IC_90_, and RID

Simulated AUC_0–24_, AUC_0–48_, AUC_0–72_, and AUC_0–96_ in maternal plasma, umbilical cord, and breast milk samples and in infant plasma samples,_,_ after the final dose of dolutegravir, following the switch to SoC (2–18 days postpartum), are summarized in Table 3, in addition to cord–maternal plasma, breast milk–maternal plasma, and infant–maternal plasma ratios (note exclusion of 1 infant owing to poor prediction of observed data). The median estimated infant half-life (range) was 37.9 (22.1–63.5) hours (n = 21).

**Table 3.** Individual Model–Predicted Areas Under the Concentration-Time Curve (AUCs) in Maternal Plasma, Breast Milk, and Infant Plasma Samples and Ratios of Breast Milk and Infant Plasma to Maternal Plasma AUCs in the 96 Hours After Dolutegravir Cessation

AUC or AUC Ratio	AUC_0–24_	AUC_0–48_	AUC_0–72_	AUC_0–96_
AUC, median (range) mg·h/L				
Maternal plasma (n = 27)	38.0 (25.6–56.4)	49.8 (31.0–83.2)	52.0 (32.0–95.1)	52.7 (32.2–100.6)
Breast milk (n = 27)	1.20 (0.71–1.95)	1.56 (0.81–2.88)	1.66 (0.82–3.29)	1.68 (0.82–3.48)
Infant plasma (n = 21)^a^	1.87 (0.62–10.3)	3.48 (1.07–17.1)	4.76 (1.34–21.3)	5.45 (1.48–24.0)
Ratio				
Breast milk–maternal plasma (n = 27)	0.033 (0.021–0.050)	0.033 (0.021–0.050)	0.033 (0.021–0.050)	0.033 (0.021–0.050)
Infant plasma–maternal plasma (n = 21)^a^	0.049 (0.018–0.26)	0.075 (0.024–0.34)	0.098 (0.028–0.41)	0.11 (0.030–0.46)

Abbreviations: AUC_0 –24_, AUC_0–48_, AUC_0–72_, and AUC_0–96_: area under the concentration-time curve, 24, 48, 72, and 96 hours after the final maternal dolutegravir dose postpartum.

^a^One infant was excluded because values were poorly predicted by the model according to individual plots and predicted pharmacokinetic parameters.

The time to the in vitro PA-IC_90_ for dolutegravir of 0.064 mg/L [14], after the final maternal dose 2–18 days postpartum, was estimated for 13 of 22 infants who were above this threshold on sampling; the median time (range) was 108.9 (18.6–129.6) hours, or 4.5 (0.8–5.4) days. In the other 9 infants, with values below the PA-IC_90_ at the time of maternal dosing, the time below this threshold could not be determined (the time to PA-IC_90_ was also not estimated for the infant whose pharmacokinetic profile was poorly defined by the model owing to its predicted trajectory). The median time postpartum (range) was 7 (3–15) days for infants with predicted times to in vitro PA-IC_90_, compared with 11 days (7–18 days) for those already below the in vitro PA-IC_90_ after the final maternal dolutegravir dose.

The average dolutegravir breast milk concentration over the 24 hours after the switch was 0.050 mg/L (range, 0.030–0.081 mg/L; n = 27), corresponding to an absolute infant dose of 2.2 µg/kg/d (1.2–3.8 µg/kg/d; n = 26; assuming 150 mL/kg/d of milk ingested [15–17]). Over the same time frame, the RID relative to that of the mother was 0.27% (range, 0.13%–0.71%; n = 26).

## DISCUSSION

The developed model satisfactorily predicted dolutegravir exposures in maternal plasma, cord blood and breastmilk, and plasma of breastfed infants participating in DolPHIN-1. Predicted maternal plasma dolutegravir AUC_0–24_ values in the third trimester and postpartum were consistent with those previously reported for the noncompartmental analysis (geometric mean [range] in the third trimester, 33.0 [21.7–53.9] vs 35.3 [19.2–67.9] mg·h/L [n = 28]; postpartum, 38.0 [25.4–56.4] vs 40.1 [22.8–59.6] mg·h/L [n = 17] [7]). Maternal dolutegravir CL/F was greater than that of another population pharmacokinetic analysis in treatment-naive, nonpregnant adults (1.50 vs 0.90 L/h) [19], resulting overall in lower AUC_0–24_ in DolPHIN-1 (eg, geometric mean, 36.1 [postpartum] vs 53.6 mg·h/L [19]; 50 mg/d).

Zhang and colleagues [19] combined data from several studies of mainly male participants but observed a 21% higher bioavailability in female participants. Despite the lower dolutegravir concentrations in DolPHIN-1, sufficient virological response was achieved and no transmissions occurred [7], suggesting that 50 mg once daily is adequate in late pregnancy. Of note, the ongoing DolPHIN-2 study (NCT03249181; n = 250; randomized in the third trimester 1:1 to efavirenz or dolutegravir-based therapy, with follow-up until 72 weeks postpartum) reported 3 early transmissions in the dolutegravir arm; these mothers quickly attained virological suppression after starting treatment. Transmission likely occurred in utero, given the low maternal viral loads at birth and early polymerase chain reaction positivity of the infants [8]. Late breastfeeding transmissions will be captured through DolPHIN-2.

There was no significant difference in dolutegravir CL/F between the third trimester and postpartum. A European clinical pharmacology network to investigate the Pharmacokinetics of newly developed ANtiretroviral agents in HIV-infected pregNAant women (PANNA) study similarly reported no significant changes between the third trimester and postpartum (n = 5) [20]. However, in both cases this may reflect insufficient time between sampling occasions for physiological changes affecting drug pharmacokinetics that occurred during pregnancy—increased gastric pH, enzymatic activity (eg, CYP3A4 and uridine 5’-diphospho-glucuronosyltransferase [UGT] 1A1), total body water and plasma volume, increased body fat composition, and decreased albumin and alpha-1-acid glycoprotein [21])—to return to the prepregnancy state (DolPHIN-1, 1–3 weeks; PANNA, 3–7 weeks).

Conversely the International Maternal Pediatric Adolescent AIDS Clinical Trials Network (IMPAACT) P1026s study demonstrated significantly lower dolutegravir AUC_0 –24_, maximum concentration, and concentration at 24 hours (29%, 25%, and 34%, respectively) in the third trimester, compared with 6–32 weeks postpartum (n = 22) [22]. Moreover, in the present analysis, none of the other evaluated covariates were significantly associated with dolutegravir model parameters. The study design was not ideal; however, at the time of study approval, dolutegravir was not recommended during pregnancy or breastfeeding by Ugandan or South African regulatory bodies, bringing a need to balance between the ideal time point and what was considered ethically acceptable. DolPHIN-2 may better elucidate pharmacokinetic disparities during and after pregnancy, using a sparse sampling schedule; these data will be incorporated into the model.

DolPHIN-1 demonstrated marked placental transfer of dolutegravir (128% that of maternal plasma), similarly to PANNA and IMPAACT P1026s [20, 22]. DolPHIN-1 was unique in evaluating transfer to breast milk, which was low, with dolutegravir concentrations 3.3% that of maternal plasma and undetectable in 88.9% of samples 46.2–50.4 hours and 100% of samples 70.8–78.3 and 94.8–98.4 hours after the switch to SoC (2–18 days postpartum). This aligns with a case report of a single mother-infant pair [23]. Covariate effects could not be investigated for the fetal (cord) or breast milk compartments of the model because inclusion of variability on associated rate constants was not supported. This is potentially owing to the sparsity of data for corresponding compartments, particularly cord, as it is only possible to take a single sample per individual, limiting the capacity of the model to differentiate between interindivdual and residual variability. However, the higher numbers and longer follow-up of DolPHIN-2 may enable the evaluation of covariates affecting breast milk transfer.

Dolutegravir infant–maternal plasma AUC ratios increased over the 3 days after cessation of maternal dolutegravir (median [range], 7 [2–18] days postpartum], suggesting delayed elimination from infants, further implied by prolonged estimated dolutegravir elimination half-lives for the infants compared with adult values (geometric mean, 40.1 hours vs 12.0 hours in adults living with HIV after 10 days of monotherapy at 50 mg/d [24]). The dolutegravir half-lives in infants were comparable to findings in IMPAACT P1026s. Similarly, based on the model-predicted half-life, dolutegravir would be expected to last 6.3–7.9 days, also consistent with IMPAACT P1026s [22].

The ontogeny of hepatic UGT enzymes (namely, UGT1A1) associated with dolutegravir metabolism, undoubtably contributes to the delayed infant clearance. Age-related changes in UGTs are not well defined, particularly in comparison with cytochrome P450. Prediction of age-related pharmacokinetic changes, through physiologically based pharmacokinetic modeling for example, requires a thorough understanding of metabolic enzyme ontogeny. Distinct developmental patterns of UGTs have been described, but with substantial variability between studies [25]. Using human liver microsomes from pediatric donors [26] or from both pediatric and adult donors [27], UGT1A1 has been shown to reach maximal adult glucuronidation activity within the early months after birth (at 3.8 months [26] and within 1 month [27], although the latter study contained few samples from donors <2 years of age [27]). No infant-associated covariates showed significant relationships with *k*_INF_; again, DolPHIN-2 may provide pertinent data.

Breastfeeding contributed relatively little to infant dolutegravir exposures after the final maternal dose before the switch to SoC. Dolutegravir is highly bound to plasma proteins (>99%), and this potentially hinders penetration into breast milk. However, dolutegravir breast milk–plasma ratio was higher than expected, which could be due to active transport into breast milk [23]. The estimated RID from breastfeeding was 0.27% that of the maternal dose, and it has been reported that medications with a RID <10% are less likely to affect the well-being of breastfeeding infants, because exposure is low or considered negligible [15, 16, 28, 29]. Regarding prevention of mother-to-child transmission, infant dolutegravir breast milk exposure is unlikely to provide adequate prophylaxis, and has been shown with nucleoside reverse-transcriptase inhibitors to present a risk for emergence of HIV drug resistance should infant infection occur [30–32]. However, the high transplacental transfer of dolutegravir could offer additional postexposure prophylaxis to some infants.

To consider the potential prophylactic coverage when dolutegravir therapy was stopped, time to the in vitro PA-IC_90_ was estimated by the model. In the 60% of infants for whom the parameter could be estimated, dolutegravir provided a median (range) of 4.5 (0.8–5.4) days of additional coverage when maternal dolutegravir was stopped 3–15 days postpartum. These infants were sampled earlier postpartum than those already below the threshold, suggesting that potential prophylactic coverage wanes with time postpartum as the transplacental “dosage” is cleared. Further simulations were performed stopping maternal dolutegravir 1, 2, and 3 weeks postpartum (data not shown).

Additional protection was predicted for a median of 2.7 and 1.4 days after stopping 1 and 2 weeks postpartum, respectively. However, the proportion of infants below the PA-IC_90_ increased from 18% at 1 week postpartum to 81% at 2 and 100% by 3 weeks. It is unlikely that mothers would stop or change therapy so quickly after childbirth. Assuming continuation of dolutegravir, given the negligible contribution from breast milk, prophylactic coverage to the infant would be achieved only in the initial days postpartum. Should infection occur, this has implications for initiation of infant ART sooner rather than later. However, a longer-term follow-up of more individuals is needed to comprehensively assess potential risk of dolutegravir resistance development before diagnosis in infants.

Assumptions were made to maximize data usage and to obtain an identifiable model. The dolutegravir dose time at the delivery visit was not known, but it was generally assumed that dosing took place in the morning, given that morning dosing was consistently recorded for third trimester and postpartum visits. Measured concentrations were also inspected to ensure a valid assumption. Time of delivery was necessary to simulate initial infant doses through prediction of cord concentration at delivery. However, the time of delivery was not always recorded. If the time of membrane rupture was recorded, although not ideal, this was used in the event of missing delivery time. Similarly, if the cord sample time rather than delivery time was recorded we assumed that delivery occurred 15 minutes before cord sampling.

The above assumptions prevented exclusion of a greater proportion of important data, particularly for the infant model. The volume of the breast milk compartment was fixed to 125 mL [16, 18], to enable identifiability; the value may be physiologically questionable, although pragmatic in the absence of more accurate and validated data. Furthermore, most of these women were attending their first antenatal appointment in late pregnancy; gestational age was estimated by the best possible means, using a combination of menstrual dates, measurement of symphyseal-fundal height, and ultrasonographic scanning, all of which are prone to error at this stage.

The developed model allowed estimation of dolutegravir disposition in multiple compartments of interest. Predicted prophylactic coverage of dolutegravir in infants as a result of transplacental passage and prolonged elimination in infants (median, 4.5 days) was generally achieved early postpartum; however, the proportion of infants without additional protection increased markedly beyond 1 week postpartum. The model will be adapted to incorporate and estimate pharmacokinetic parameters for DolPHIN-2 to further elucidate dolutegravir pharmacokinetics during pregnancy and over a longer postpartum period.

## Supplementary Data

Supplementary materials are available at *Clinical Infectious Diseases* online. Consisting of data provided by the authors to benefit the reader, the posted materials are not copyedited and are the sole responsibility of the authors, so questions or comments should be addressed to the corresponding author.

ciaa1861_suppl_Supplementary_Materials

## Notes

***Acknowledgments.*** The authors thank the mothers and families who participated in DolPHIN-1, the staff from the centers taking part in the trial, and all the research staff involved. The authors particularly acknowledge additional members of the DolPHIN-1 study team: Ritah Nakijoba, Isabella Kyohairwe, Johnson Magoola and Emmanuel Ssempija.

***Financial support.*** This work was supported by ViiV Healthcare (provision of study drug and grant 205785 to S. K.) and by the Wellcome Trust (clinical postdoctoral training fellowship WT104422MA to C. W.).

***Potential conflicts of interest.*** C. H. reports shares with GlaxoSmithKline, outside the submitted work. C. W. report grants from ViiV Healthcare (investigator-led academic funding for the clinical trial), during the conduct of the study. S. K. has received research support/grants from ViiV for DolPHIN studies and grants from Gilead for Drug interactions Web site support, outside the submitted work, and has received research support from Merck and Janssen and speaker honoraria from ViiV. M. L. has received research grants from ViiV and nonfinancial support (study drug) during the conduct of the study; and grants and personal fees from Janssen, grants from Merck, and personal fees, grants, and nonfinancial support from Mylan, outside the submitted work. All other authors report no potential conflicts. All authors have submitted the ICMJE Form for Disclosure of Potential Conflicts of Interest. Conflicts that the editors consider relevant to the content of the manuscript have been disclosed.

Presented in part: Conference on Retroviruses and Opportunistic Infections (CROI 2019), Seattle, Washington, 4-7 February 2019 (abstract 0757); 20th International Workshop on Clinical Pharmacology of HIV, Hepatitis and Other Antiviral Drugs, Noordwijk, the Netherlands, 14–16 May 2019 (abstract 9).
