# Supplementary material for: Infant Exposure to Dolutegravir Through Placental and Breast Milk Transfer: A Population Pharmacokinetic Analysis of DolPHIN-1
Source: Clin Infect Dis. 2020 Dec 21;73(5):e1200–7. doi: 10.1093/cid/ciaa1861 (PMC8423479; doi:10.1093/cid/ciaa1861)
Supplement: ciaa1861_suppl_Supplementary_Materials [file ciaa1861_suppl_supplementary_materials.docx]

**Supplementary Material**

**Methods**

***Population Pharmacokinetic Modelling***

Analytical solutions were used in the $PRED subroutine section of NONMEM to estimate model parameters implementing the LAPLACIAN estimation method. Infant dolutegravir parameters were determined through a sequential approach whereby parameters of the maternal model were fixed to the individual Bayesian estimates for the analysis of infant data (infant model) applying ordinary differential equations (ODEs) in the $DES subroutine of NONMEM. Infants with a recorded date and time of delivery were included and their initial dolutegravir ‘*dose*’ estimated by conversion of the cord concentration at time of delivery to an amount in milligrams by multiplication of the concentration and maternal central volume of distribution. This amount is akin to an infusion that has occurred over the weeks prior to birth and the only other dolutegravir the infants received was through breastmilk. Feeding times were not known however, during model development emptying of the breastmilk compartment to mimic a feed was assessed after pharmacokinetic sampling but this did not impact the fit and was not included in the final model.

Samples below the assay LLQ (BLQ) were included in the models according to one of two approaches. If the proportion of samples BLQ from a particular compartment was <10% they were included as LLQ/2 (0.005 mg/L) however, if the proportion was >10% the M3 method was implemented, which incorporates the likelihood of being BLQ into the model [1-3].

*Covariate analysis*

The following covariates were assessed: pregnancy (third trimester *vs.* postpartum), mode of delivery (vaginal *vs.* caesarean section), bodyweight, age and weeks postpartum for the maternal model; and birth weight, body surface area (BSA), postnatal, gestational and postmenstrual age and sex for the infant model. Univariable covariate analysis was performed initially and progressed to multivariable analysis if multiple covariates were significantly associated with model parameters. Goodness-of-fit was assessed by means of graphical and statistical methods. A forwards-inclusion backwards-elimination method was used to assess covariates in the model. A decrease in the minimal objective function value (OFV; -2 log likelihood) of at least 3.84 units was required to accept a model with an additional parameter (*p*=0.05, χ^2^ distribution, 1d.f.). Once significant covariates were incorporated, backwards elimination was performed and biologically plausible covariates producing an increase in OFV of ≥10.83 units (*p*=0.001, χ^2^ distribution, 1d.f.) were retained.

*Model evaluation*

To evaluate the models, visual predictive checks (VPC) with or without prediction-correction (pcVPC [4]; correcting for inclusion of significant covariates and/or different doses per individual) were generated from 1000 simulations of the dataset. The VPCs were performed using Perl-speaks-NONMEM (PsN; version 3.4.2) [5]; Pirana software (version 2.9.0) acted as an interface between PsN and NONMEM and tracked model development. VPC plots were developed using Xpose4 in RStudio (version 1.1.383) [6].

**Supplementary Figure 1** Schematic of the DolPHIN-1 study design and dolutegravir pharmacokinetic sampling schedule (note that efavirenz pharmacokinetic evaluation was not performed).

DTG: dolutegravir; EFV: efavirenz; TDF: tenofovir disoproxil fumarate; FTC: emtricitabine; 3TC: lamivudine; PK: pharmacokinetic

**Supplementary Figure 2** Dolutegravir goodness-of-fit plots for (**a**) maternal plasma (n=28 patients, 533 concentrations), (**b**) umbilical cord (n=16 patients, 16 concentrations), (**c**) breastmilk (n=26 patients, 49 concentrations) and (**d**) infant plasma (n=22, 65 concentrations) with observed concentrations (dependent variable; DV) *vs.* population predictions (PRED) in the left panel and DV *vs.* individual predictions (IPRED) in the right panel. The fine line describes the line of unity and the bold line the line of regression.

(**b**)

(**a**)

(**d**)

(**c**)

|  |  |
| --- | --- |
|  |  |
|  |  |
|  |  |

*Due to the use of the M3 method, conditional weighted residuals (CWRES) vs. PRED and time cannot be plotted since CWRES is not determined for those patients with at least 1 sample below the limit of quantification (BLQ) in any compartment. Furthermore, the M3 method does not produce PRED concentrations but a probability of being BLQ; therefore, BLQ samples have been removed for plotting purposes.*

**References**

1. Ahn JE, Karlsson MO, Dunne A, Ludden TM. Likelihood based approaches to handling data below the quantification limit using NONMEM VI. J Pharmacokinet Pharmacodyn **2008**; 35(4): 401-21.

2. Beal SL. Ways to fit a PK model with some data below the quantification limit. J Pharmacokinet Pharmacodyn **2001**; 28(5): 481-504.

3. Bergstrand M, Karlsson MO. Handling data below the limit of quantification in mixed effect models. AAPS J **2009**; 11(2): 371-80.

4. Bergstrand M, Hooker AC, Wallin JE, Karlsson MO. Prediction-corrected visual predictive checks for diagnosing nonlinear mixed-effects models. AAPS J **2011**; 13(2): 143-51.

5. Lindbom L, Pihlgren P, Jonsson EN. PsN-Toolkit--a collection of computer intensive statistical methods for non-linear mixed effect modeling using NONMEM. Comput Methods Programs Biomed **2005**; 79(3): 241-57.

6. Jonsson EN, Karlsson MO. Xpose--an S-PLUS based population pharmacokinetic/pharmacodynamic model building aid for NONMEM. Comput Methods Programs Biomed **1999**; 58(1): 51-64.
